# Functional Calcium Binding Peptides from Pacific Cod (*Gadus macrocephalus*) Bone: Calcium Bioavailability Enhancing Activity and Anti-Osteoporosis Effects in the Ovariectomy-Induced Osteoporosis Rat Model

**DOI:** 10.3390/nu10091325

**Published:** 2018-09-18

**Authors:** Kai Zhang, Bafang Li, Qianru Chen, Zhaohui Zhang, Xue Zhao, Hu Hou

**Affiliations:** College of Food Science and Engineering, Ocean University of China, No.5, Yu Shan Road, Qingdao 266003, China; decond@163.com (K.Z.); bfli@ouc.edu.cn (B.L.); qianru.chen@outlook.com (Q.C.); zhangzhh@ouc.edu.cn (Z.Z.); zhaoxue@ouc.edu.cn (X.Z.)

**Keywords:** calcium binding peptide, peptide-calcium complex, intestinal calcium absorption, calcium bioavailability, anti-osteoporosis activity

## Abstract

Calcium binding peptides from Pacific cod (*Gadus macrocephalus*) bone have attracted attention due to their potential effects on bone health. In this study, calcium binding peptides (CBP) were prepared from Pacific cod bone by trypsin and neutral protease. Ultraviolet spectra, circular dichroism (CD), and Fourier transform infrared spectroscopy (FTIR) revealed that carboxyl and amino groups in CBP could bind to Ca^2+^, and form the peptide-calcium complex (CBP-Ca). Single-pass intestinal perfusion (SPIP) experiments indicated that the intestinal calcium absorption was significantly enhanced (*p* < 0.01) in CBP-Ca treated Wistar rats. The anti-osteoporosis activity of CBP-Ca was investigated in the ovariectomized (OVX) Wistar rat model. The administration of CBP-Ca significantly (*p* < 0.01) improved the calcium bioavailability, trabecular bone structure, bone biomechanical properties, bone mineral density, and bone mineralization degree. CBP-Ca notably (*p* < 0.01) increased serum calcium, however, it remarkably (*p* < 0.01) reduced the levels of osteocalcin (OCN), bone alkaline phosphatase (BALP), tartrate-resistant acid phosphatase isoform 5b (TRAP5b), and C-telopeptide of type I collagen (CTX-1) in serum. Results suggested that the cod bone derived CBP could bind with calcium, improve the intestinal calcium absorption, calcium bioavailability, and serum calcium, then reduce the bone turnover rate, and thus ameliorate osteoporosis.

## 1. Introduction

Osteoporosis is a prevalent chronic disease that may result in fracture [1,2]. It has been characterized by the undesirable high bone turnover rate, the loss of bone mass, and the malabsorption of calcium [3]. Previous studies suggested that the osteoporosis and bone loss could be ameliorated by restoring intestinal calcium absorption [4], and many pharmacological compounds (such as strontium ranelate, teriparatide, raloxifene, alendronate, hormone melatonin, etc.) have been applied in this area [3,4,5]. However, these medicines do have some limitations such as undesired side effects, drug resistances, and high prices [3,4,6]. Therefore, health professionals have paid much attention to both safety and economical food-derived compounds to enhance intestinal calcium absorption [4], especially the calcium binding peptides [7,8].

Calcium binding peptides could interact with Ca^2+^ during the formation of the peptide-calcium complex [8], which was believed to be a suitable candidate for calcium supplementation [9]. In the recent decade, calcium binding peptides and the peptide-calcium complex have attracted great attention and many food proteins have been explored to prepare and isolate calcium binding peptides, including tilapia protein [10], Antarctic krill protein [11], Pacific cod skin protein [12], whey protein [13], duck egg white [14], etc. Furthermore, previous studies reported that fishbone derived peptides also possess high calcium binding activity and can improve calcium bioavailability [15,16]. Thus, this provides a possibility for their anti-osteoporosis activities [17].

Fishbone has often been discarded directly as the major by-product during aquatic processing [18]. However, it contains a high protein content (about 30 g protein per 100 g dry fishbone) [19]. This makes fishbone an excellent resource of proteins and peptides [16,18]. Therefore, many researches have been devoted to the utilization of fishbone derived bioactive peptides [18], especially the calcium binding peptides and peptide-calcium complex [8,15]. However, studies on the anti-osteoporosis activity of fish bone peptide-calcium complex have been rare, as most of them have only paid attention to calcium bioavailability.

The main purpose herein was to investigate the calcium bioavailability enhancing activity and the anti-osteoporosis effects of Pacific cod bone derived calcium binding peptides. In the present study, calcium binding peptides (CBP) were prepared from Pacific cod bone by trypsin and neutral protease. Then the peptide-calcium complex (CBP-Ca) was prepared and characterized. The intestinal calcium absorption of CBP-Ca was investigated by using the single-pass intestinal perfusion (SPIP) experiment. Subsequently, the calcium bioavailability and anti-osteoporosis effects of CBP-Ca were studied utilizing the ovariectomized rat model. Parameters including calcium apparent absorption rate, calcium retention rate, bone mass, bone mineral density, bone biomechanical properties, bone histomorphometry, and bone turnover markers were investigated.

## 2. Materials and Methods

### 2.1. Materials

Pacific cod (*Gadus macrocephalus*) bone gelatin was prepared according to our previous report [16]. Neutral protease and trypsin were obtained from Pangbo Biotech Co., Ltd. (Nanning, China). The 4-(2-hydroxyethyl)-1-piperazine-ethanesulphonic acid (HEPES), deuteroxide (D_2_O), and raloxifene were purchased from Sigma-Aldrich (St. Louis, MO, USA). Commercial kits for serum calcium (Ca), phosphorus (P), osteocalcin (OCN), bone alkaline phosphatase (BALP), and tartrate-resistant acid phosphatase isoform 5b (TRAP5b) were provided by Nanjing Jiancheng Bioengineering Institute (Nanjing, China). ELISA assay kit of C-telopeptide of type I collagen (CTX-1) was obtained from Shanghai Enzyme-linked Biotechnology Co., Ltd. (Shanghai, China). All other chemicals and reagents were of analytical purity.

### 2.2. Preparation of CBP

The peptides with the highest binding affinity for calcium ions were optimized by calcium binding assay. Finally, the cod bone gelatin solution (2.5% *w*/*w*) was hydrolyzed by 0.5% trypsin and neutral protease (pH 7.2, 52 °C, 90 min) for preparing the calcium binding peptides. The CBP was lyophilized and stored at −20 °C until use. The calcium binding assay was performed according to the method described previously [20].

### 2.3. Amino Acid Profile

The amino acid composition of CBP was determined according to the method described by Liu and co-workers [13]. Samples were hydrolyzed in 6 M HCl (130 °C, 4 h), then an amino acid analyzer (Hitachi, Tokyo, Japan) was adopted to analyze the hydrolysates [13].

### 2.4. Preparation of CBP-Calcium Complex (CBP-Ca)

CaCl_2_ (11.0 g) was mixed with 500 mL 6% (*w*/*v*) CBP solution (pH 7.0), and incubated at 50 °C for 1 h. The resulted solution was subsequently mixed with 8 times the volume of absolute ethanol, precipitated for 30 min, and then centrifuged at 7000× *g* for 15 min to remove the free Ca^2+^. Finally, the CBP-Ca sedimentation was collected, lyophilized, and stored at −20 °C until use.

### 2.5. Structural Characterization of CBP and CBP-Ca in Solution

#### 2.5.1. UV Absorption

The UV absorption spectrum was recorded by a UV-spectrophotometer (Shimadzu UV-2550, Kyoto, Japan). The sample solution (0.2 mg/mL) was placed into a quartz cell with a path length of 1 cm. The UV spectrum was measured by scanning the wavelength from 190 to 400 nm at a scan speed of 2 nm/s with an interval of 1 nm.

#### 2.5.2. Circular Dichroism Analysis

The circular dichroism (CD) spectra of CBP and CBP-Ca were recorded at 25 °C using a Jasco J-815 spectrophotometer (Japan Spectroscopic Company, Tokyo, Japan). The CBP (1.0 mg/mL) solution was scanned in the presence of 20 mM HEPES buffer (pH 7.0). The CBP-Ca solution was prepared by adding 3 μM CaCl_2_ into 1.0 mg/mL CBP solution (pH 7.0). The mixed solution was incubated at 50 °C for 1 h before CD analysis. Each scan of the sample was executed after subtracting the spectrum of 20 mM HEPES buffer.

#### 2.5.3. Fourier Transform Infrared (FTIR) Spectroscopy Measurement

FTIR measurement was implemented using a Nicolet 200SXV infrared spectrophotometer (Thermo-Nicolet Co., Madison, WI, USA) at 25 °C, according to the method reported by Nara et al. [21]. To obtain reliable infrared spectra in solution, the CBP and CBP-Ca were completely deuterated by dissolving in deuteroxide at 65 °C for 2 h, respectively. The deuterated samples were then lyophilized and collected for FTIR analysis. The peptide concentration of each sample for FTIR measurement was 20 mg/mL. Each FTIR spectrum was scanned at a data acquisition rate of 2 cm^−1^ per point and normalized by the Omnic 6.0 software (Thermo-Nicolet Co., Madison, WI, USA).

### 2.6. In Situ Single-Pass Intestinal Perfusion (SPIP) Study

#### 2.6.1. Animals

All animal procedures were approved by the ethical committee of animal research in the Ocean University of China, and complied with the requirements of the National Act on the use of experimental animals (China). Twelve male Wistar rats (210 ± 10 g; Licensed ID: SCXK 2014-0007) arrived at the animal chamber at least 7 days before the perfusion study, with free access to distilled water and pelleted AIN-93 diet. They were randomly assigned into two groups (*n* = 6), namely group A (CaCl_2_) and group B (CBP-Ca). Prior to the SPIP experiment, all rats were kept with overnight fasting but free access to water.

#### 2.6.2. Single-Pass Intestinal Perfusion (SPIP) Experiment

**Figure 1:**
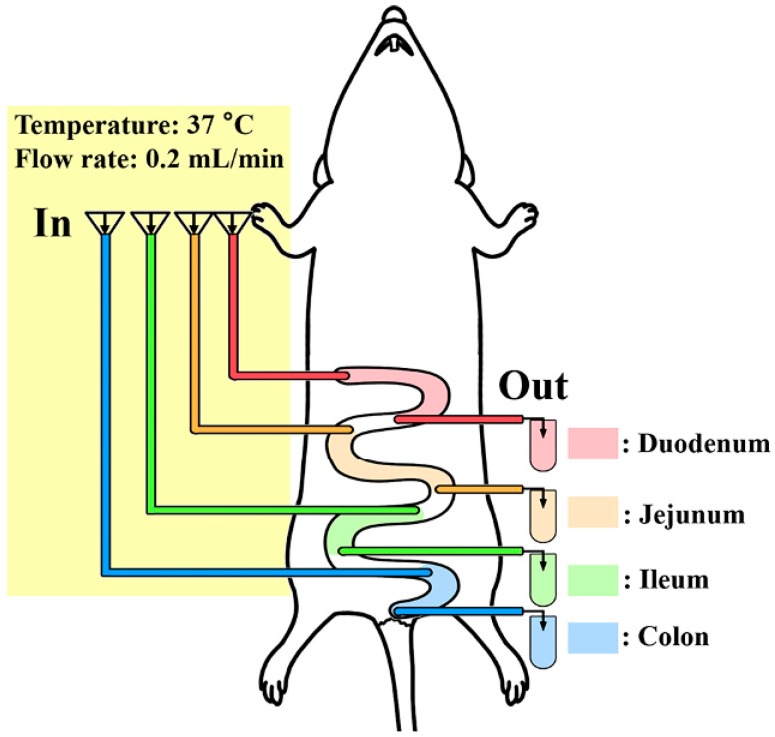
In situ single-pass intestinal perfusion (SPIP) system.

The SPIP experiment was conducted according to previous reports [22,23]. Rats were anesthetized with sodium pentobarbital (25 mg/kg) and fixed upon a heating pad (37 °C) to keep the body temperature. The abdominal incision was opened along the belly line (4–5 cm). Four intestine segments (duodenum, jejunum, ileum, and colon) were identified and carefully exposed for about 10 cm, followed the method reported previously [23]. Each segment was flushed gently with physiological saline to remove the residues, and cannulated with a polypropylene tube (diameter: 4 mm) on the proximal and distal end, respectively. Each inlet tube was then fitted together with a peristaltic pump, and the outlet tube was connected to a collecting vial. The surgical area was covered with absorbent cotton saturated with physiological saline (37 °C) to avoid dehydration (Figure 1).

Prior to the perfusion study, Krebs–Ringer buffer (KRB) pH 7.4 was perfused for 30 min to attain a steady system. After reaching the steady-state, rats of group A were perfused with 10 mM CaCl_2_ (dissolved in KRB), and the rats of group B were perfused with CBP-Ca solution (containing 10 mM Ca^2+^, dissolved in KRB). In order to simulate real in vivo condition as much as possible, the CaCl_2_ and CBP-Ca were pre-treated under simulated gastric conditions. The flow rate was 0.2 mL/min, and all solutions used in the SPIP studies were pre-warmed at 37 °C and heated in a water bath (37 °C) throughout the experiment. The outflow liquid of each outlet tube was quantitatively collected at 15 min intervals for 90 min. The calcium content was determined by a flame atomic absorption spectrometry (FAAS). Finally, the rats were euthanized, the length and radius of each perfused intestinal segment were measured.

#### 2.6.3. Data Analysis and Equations

The calcium percent absorption, calcium absorption rate constant (*Ka*), and calcium effective permeability (*P_eff_*) were calculated using Equations (1)–(3), respectively.

(1) $$Calcium\;percent\;absorption\;\left(\%\right)=\left(1-\frac{C_{out}\times Q_{out}}{C_{in}\times Q_{in}}\right)\times 100\%$$

(2) $$K_{a}=\left(1-\frac{C_{out}\times Q_{out}}{C_{in}\times Q_{in}}\right)\times \frac{v_{in}}{\pi r^{2}l}$$

(3) $$P_{eff}=\frac{-v_{in}\times \ln\left(\frac{C_{out}\times Q_{out}}{C_{in}\times Q_{in}}\right)}{2\pi rl}$$

where *C_out_* and *C_in_* are the respective calcium concentration in the outlet and inlet perfusate, *Q_out_* and *Q_in_* are the respective volumes of outlet and inlet perfusate, *v_in_* is the inlet flow rate of perfusate, *r* and *l* represent the radius and length of perfused intestinal segment after the SPIP experiment.

### 2.7. Calcium Bioavailability and Anti-Osteoporosis Activity of CBP-Ca

#### 2.7.1. Animals and Treatments

Forty-two female Wistar rats aged 2 months (220 ± 10 g; Licensed ID: SCXK 2014-0007) were purchased from Pengyue Laboratory Animal Breeding Center (Jinan, China) and acclimated for 7 days to a controlled environment (23 ± 1 °C, 12–12 h light–dark cycle). All rats were individually housed in stainless cages and allowed free access to pelleted AIN-93 diet and deionized water. The diet was adjusted to either normal calcium diet (12.5 g CaCO_3_/kg diet) or low calcium diet (2.5 g CaCO_3_/kg diet). All animal experiments were approved by the ethical committee of animal research in the Ocean University of China.

Anaesthetized animals were randomly given either bilateral laparotomy (sham ovariectomy operated) as SHAM group (*n* = 7), or bilateral ovariectomy (OVX) to establish the osteoporosis model (*n* = 35) [24]. The content of serum β-estradiol (E_2_) was determined routinely to confirm the establishment of the osteoporosis model. Compared with the SHAM group, rats with a significant decrease in serum E_2_ content were confirmed as a successful osteoporosis model. Thirty-five osteoporosis model rats were randomly allocated into five groups (*n* = 7/group): OVX group (treated with normal physiological saline as a negative control group, 7 mL/kg/body weight), RAL group (treated with raloxifene as the positive control model, 3 mg/kg/body weight), CaCO_3_ treated group (Ca^2+^ content: 200 mg/kg/body weight), CBP-Ca-L treated group (Ca^2+^ content: 100 mg/kg/body weight) and CBP-Ca-H group (Ca^2+^ content: 200 mg/kg/body weight). The SHAM group was treated with 7 mL/kg/body weight normal saline. According to this protocol, each rat was intragastrically administrated with a volume of 10 mL/kg/body weight once a day for 90 days. To maintain the same calcium intake, the SHAM, OVX, and RAL groups were fed with a normal calcium diet, but the other groups were fed with a low calcium diet.

#### 2.7.2. Calcium Bioavailability of CBP-Ca

To determine the calcium bioavailability, rats were placed in individual metabolic cages for the last 3 days of the experiment. Parameters including calcium apparent absorption (mg/day), calcium apparent absorption rate (%), calcium retention (mg/day), and calcium retention rate (%) were determined according to previous study [16].

#### 2.7.3. Sampling and Analytical Methods

All rats were fasted for 12 h and anaesthetized, after the 90 days experiment. The blood was collected from the abdominal aorta and centrifuged to separate the serum. Then the contents of Ca, P, OCN, and CTX-1, as well as the activities of BALP and the TRAP5b in the serum were determined using commercial kits. Rats were then sacrificed, and their tibias and femurs were dissected. Right femurs were weighed, and their calcium contents were determined according to the method described previously [16]. Left femurs were selected to evaluate the maximum loads using the three-point bending method [25]. Right tibias were collected for the determinations of bone mineral density (BMD) and bone mineralization degree [26]. Left tibias were fixed with 10% methanol and decalcified by EDTA, then the bone histomorphology was analyzed by the hematoxylin–eosin (HE) staining method [24].

### 2.8. Statistical Analysis

Statistical significance between two treatments was assessed by Student’s *t*-tests using an SPSS 18.0 software (SPSS Inc., Chicago, IL, USA). All data were expressed as mean ± standard deviation (SD), and differences were considered statistically significant if *p* < 0.05.

## 3. Results and Discussion

### 3.1. Calcium Binding Activity and Amino Acid Composition of CBP

The calcium binding activity of CBP was determined as 0.43 ± 0.09 μg/mg. It is similar to that of the Alaska pollack skin derived calcium binding peptide (0.55 μg/mg) [20]. Amino acid analysis of CBP (shown in Table 1) suggested that it was rich in Gly, Pro, Hyp, Ala, Glu, Ser, Asp, Arg, and His. The glycine and imino acids (Pro and Hyp) contents of CBP were 322 and 178 residues/1000 residues, respectively. Similar results were found in enzymatic hydrolysis of tilapia scale collagen [25] and sea bream bones collagen peptides [27]. In addition, previous reports indicated that the carboxyl groups of Asp and Glu and the amino groups in Arg and Lys were responsible for the calcium binding activity of the peptides [8,10]. The Glu, Asp, Arg and Lys contents in CBP were 97, 84, 46, and 14 residues/1000 residues, respectively. Liu, et al. reported that wheat germ protein derived calcium binding peptides are rich in Glu, Asp, and Arg [13]. Hou et al., suggested that the acidic amino acids, as well as basic amino acids, contribute to the calcium binding activity of peptides [11]. These are consistent with our study. Results suggested that the specific amino acid residues might provide suitable calcium binding sites for CBP, therefore, contribute to its calcium binding activity.

### 3.2. Structural Differences between CBP and CBP-Ca in Solution

#### 3.2.1. UV Absorption Spectrum of CBP and CBP-Ca

**Figure 2:**
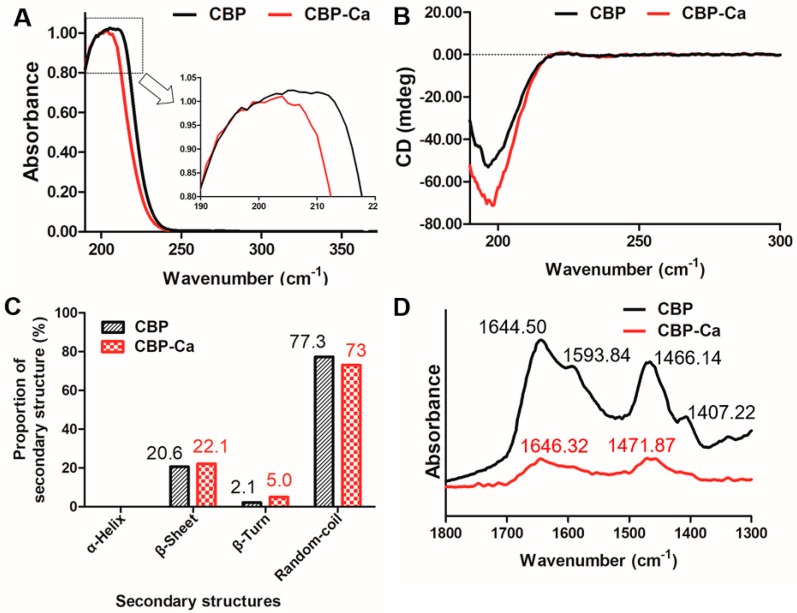
Structural differences of calcium binding peptide (CBP) and CBP-Ca in solution. (**A**) UV absorption spectra; (**B**) circular dichroism spectroscopic data; (**C**) proportions of secondary structures; (**D**) Fourier transform infrared spectroscopy analysis.

Figure 2A shows that the UV spectra of CBP changed before and after the presence of calcium. After cooperating with calcium, the absorption peak of CBP shifted from 206 nm to 204 nm, and a small peak arose in the UV absorption curve of CBP-Ca. A similar phenomenon was also observed by Zhao, et al. in the Gly-Tyr and Gly-Tyr-Ca [28]. This indicated that CBP could bind with Ca^2+^ and form CBP-Ca.

#### 3.2.2. Secondary Structure of CBP and CBP-Ca in Solution

CD analysis was implemented to investigate the secondary structure differences between CBP and CBP-Ca [29]. Figure 2B showed the full CD spectra (190 to 300 nm) of CBP and CBP-Ca. The CD spectrum of CBP changed significantly after calcium was presented. The random coil was the predominant structure, followed by β-sheets and β-turns, while the α-helix structure was absent in both CBP and CBP-Ca (shown in Figure 2C). Compared with CBP, the secondary structure composition of CBP-Ca changed dramatically: the β-turn content doubled (from 2.1% to 5.0%), the proportion of β-sheet increased from 20.6% to 22.1%, while the percentage of random-coil decreased from 77.3% to 73%. These results revealed that a compact structure was formed after Ca^2+^ interacted with CBP. A similar result was reported previously [16].

#### 3.2.3. Fourier Transform Infrared (FTIR) Spectra of CBP and CBP-Ca

As a powerful instrument for investigating the peptide structures [30], the FTIR technique was employed for the further study of the mechanisms underlying peptide-calcium reactions. The FTIR spectra of CBP and CBP-Ca in D_2_O solution were normalized against the peak intensity, and the major vibration bands (1800 to 1300 cm^−1^) were identified and associated with the main peptide groups (Figure 2D).

The amide-I, amide-II, and –COOH stretching vibration bands were the most valuable and informative infrared bands in the investigations of peptide secondary structures in solution [30,31]. The infrared absorbance band at 1644.50 and 1466.14 cm^−1^ of CBP could be attributed to the amide-I band (C=O stretching vibration) and amide-II band (mainly caused by N–H bending vibration), respectively. In the infrared spectrum of CBP-Ca, these bands shifted to 1646.32 and 1471.87 cm^−1^, respectively. Meanwhile, their intensities decreased significantly. These results indicated that the carboxylate groups and amino groups in CBP contributed to the calcium binding reaction [16,28]. Previous researches suggested that the infrared absorption of amino acid side chains provide a wealth of information, which could be used to research the peptide reactions mechanism [31]. In the CBP spectrum, the infrared peak at 1593.84 and 1407.22 cm^−1^ representing the antisymmetric stretching vibration and the symmetric stretching vibration of side chains –COOH in CBP, respectively [30,31]. These –COOH variation bands disappeared in the presence of Ca^2+^, indicating that –COOH interacted with calcium ions, therefore, transformed into –COO–Ca [13,16].

Results demonstrated that the Ca^2+^ binds to CBP via interactions with amino N atoms and the carboxyl O atoms in CBP. Chen et al. [25] reported that calcium ions could bind to the carbonyl group and the amino group in tilapia derived calcium binding peptides. Similarly, Zhao et al. suggested that –NH_3_ and –COOH contribute to the calcium binding activity of calcium binding peptides [28,32].

### 3.3. CBP-Ca Improved the Rat Intestinal Calcium Absorption

**Figure 3:**
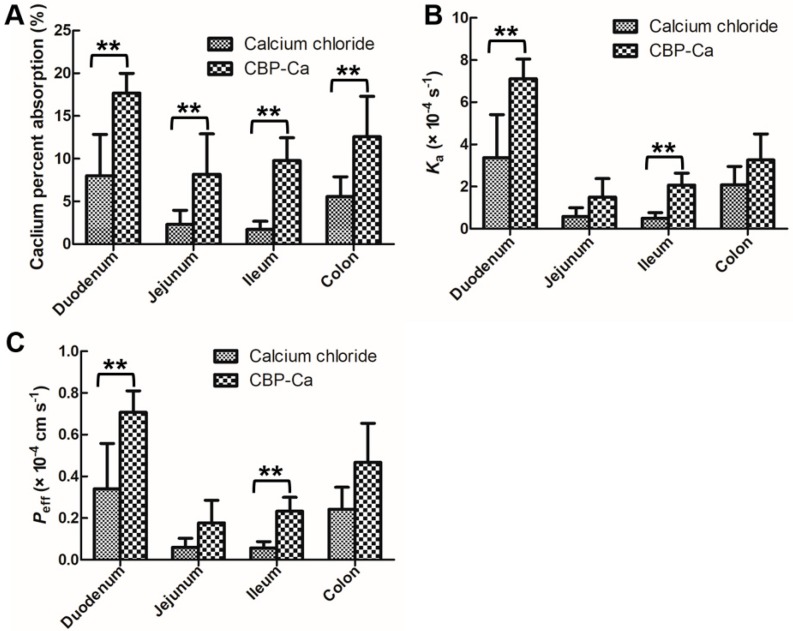
Effects of CBP-Ca on intestinal calcium absorption. The calcium percent absorption (**A**), calcium absorption rate constant (*Ka*) (**B**), and calcium effective permeability (*P_eff_*) (**C**) comparisons of CaCl_2_ and CBP-Ca in four different intestinal segments. All values are expressed as the mean ± SD. (each *n* = 6). ** *p* < 0.01.

The in situ single-pass intestinal perfusion (SPIP) experiment system (see Figure 1), which provides the intact intestinal barrier, functional blood circulation, as well as normal secretion of enzymes and transporters [33], could be used with precision in the forecasting of intestinal calcium absorption in the human body [22,33]. Calcium absorption percent, calcium absorption rate constant (*Ka*), and calcium effective permeability (*P_eff_*) were determined in four intestine segments utilizing the SPIP study (see Figure 3A–C).

Duodenum and ileum are the two most important intestine segments in transcellular and paracellular calcium transport [34,35], Figure 3C showed that the calcium *P_eff_* × 10^−4^ values of group A (CaCl_2_ treated) were 0.341 ± 0.217 and 0.0561 ± 0.031 cm s^−1^ in duodenum and ileum, respectively. In group B (CBP-Ca treated), they were increased to 0.708 ± 0.103 and 0.233 ± 0.067 cm s^−1^, respectively. A similar trend was seen in the *Ka* values (see Figure 3B). Meanwhile, a significant increase in calcium absorption percent between the two groups was also observed in each intestine segment (Figure 3A).

The high calcium permeability of CBP-Ca indicated that, in combination with CBP, the calcium transported rapidly into enterocytes [33,36]. Thus, the calcium in the peptide-bound state is superior to the free calcium ions in term of intestinal calcium absorption. These results suggested that the CBP-Ca could improve the intestinal permeability and absorption of calcium [23]. Previous studies suggested that the peptide-calcium complex is a superior candidate to those of ionized calcium (such as calcium chloride, calcium carbonate, calcium lactate, and calcium gluconate) for promoting calcium absorption in the intestinal tract [8,9]. This is consistent with our results.

### 3.4. CBP-Ca Improved the Calcium Bioavailability of OVX Rats

**Figure 4:**
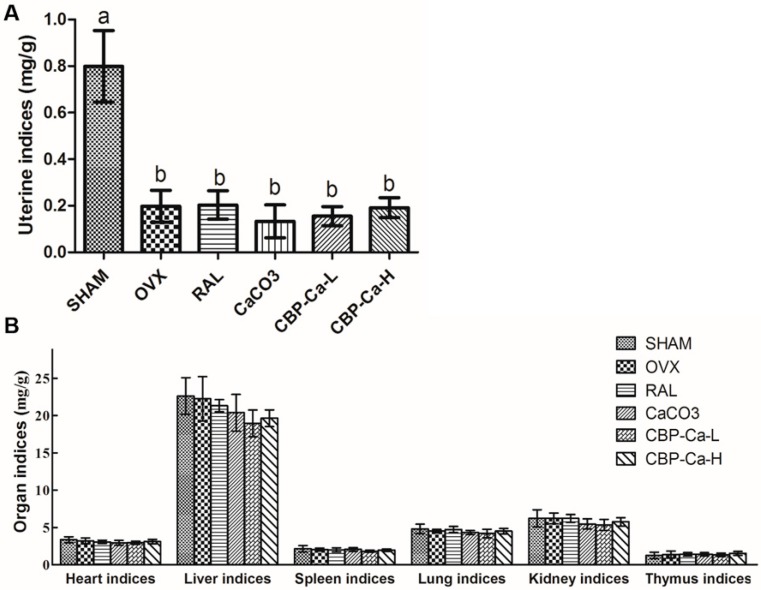
Effects of CBP-Ca on the uterine indices (**A**) and organ indices of different internal organs (**B**). All values are presented as mean ± SD (*n* = 7/group). Different lowercases letters representing significant differences (*p* < 0.05).

The uterine index of the OVX group was significantly lower than that of the SHAM group, caused by OVX-induced estrogen deficiency (Figure 4A). This indicated that the OVX model had been established successfully. In addition, part B of Figure 4 showed that there were no statistical differences of other organic indices between each group. It suggested that the administration of CBP-Ca does not affect organ health.

As Table 2 shows, there was no statistical difference in calcium intake between each group (expect for the CBP-Ca-L group). The fecal Ca of ovariectomized (OVX) and CaCO_3_ groups (34.63 and 29.13 mg) were significantly higher than that of SHAM (19.57 mg) group. This could be explained by the OVX-induced calcium malabsorption [3]. However, there were no statistical differences in fecal Ca between the SHAM (19.57 mg), RAL (19.43 mg), and CBP-Ca-H (18.16 mg) groups.

In addition, the calcium retention rate of OVX group was 38.72%, remarkably lower than that of the SHAM group (63.33%, *p* < 0.01). Whereas, the calcium retention rate was notably increased via the administration of raloxifene or CBP-Ca, compared to CaCO_3_. Meanwhile, the calcium apparent absorption rate follows a similar trend. These results indicated that the calcium absorption of OVX rats could be significantly improved by the administration of CBP-Ca (*p* < 0.01), and the CaCO_3_ treatment has no effect on the restoration of impaired calcium absorption in OVX rats (*p* > 0.05). Peng et al. suggested that fish bone peptides can increase the calcium bioavailability in rats [16]. In another study, Chen et al. reported that calcium absorption was improved by tilapia scale derived peptides [25]. These findings are in accordance with our results in the present study. According to the SPIP results shown in Figure 3, the intestinal permeability and intestinal absorption of calcium was significantly improved by CBP-Ca. The comprehensive analysis of the results of Figure 3 (SPIP experiments data) and Table 2 (calcium bioavailability results) suggested that CBP-Ca could improve the intestinal permeability and absorption of calcium, thus enhance calcium bioavailability.

### 3.5. CBP-Ca Has Protective Effects in Bone Microarchitecture of OVX Rats

**Figure 5:**
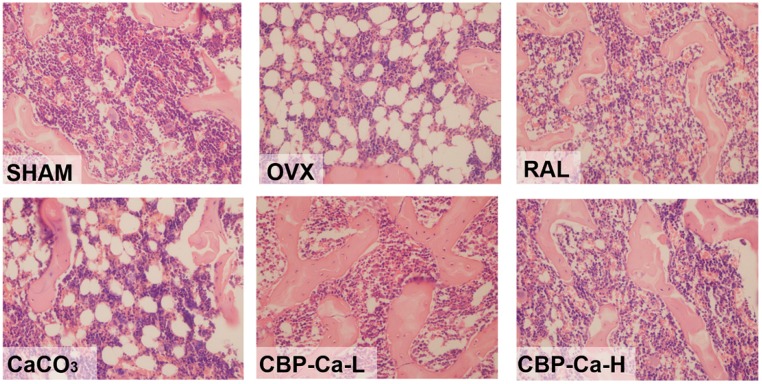
Effect of CBP-Ca on bone (left tibias) histomorphometry. Left tibias were dissected, eliminated the adhering tissues, fixed with methanol, decalcified, stained with the hematoxylin–eosin (HE) staining technique, and observed under an Olympus BX41 optical microscope (Olympus, Tokyo, Japan, magnification ×40).

The histomorphometry changes of tibias were investigated by HE staining. As shown in Figure 5, the SHAM group presented with well-formed and competent trabecular bone, which was normal in compactness and density. However, the trabecular bone in the tibia of OVX group rats was poorly observed. In addition, these trabecular structures were significantly thinning and widely separated, revealing a severe degree of osteoporosis in the OVX rats. Similar phenomena were also observed in previous studies [24,37]. Rats in CaCO_3_ and CBP-Ca-L groups have a mild degree of osteoporosis as moderate recovery of trabecular bone. Meanwhile, the rats treated by raloxifene and high dose CBP-Ca exhibited significant recovery with nearly completely recovered trabecular structure and density. These reults suggested that the OVX induced osteoporosis was slightly alleviated in the CaCO_3_ and CBP-Ca-L groups, and significantly recovered by raloxifene and high dose CBP-Ca. However, the daily Ca^2+^ intake of CBP-Ca-H group was equal to that of the CaCO_3_ group (200 mg/kg/body weight). This indicated that CBP-Ca is superior to CaCO_3_ in bone restoration.

### 3.6. CBP-Ca Enhanced Bone Properties in OVX Rats

**Figure 6:**
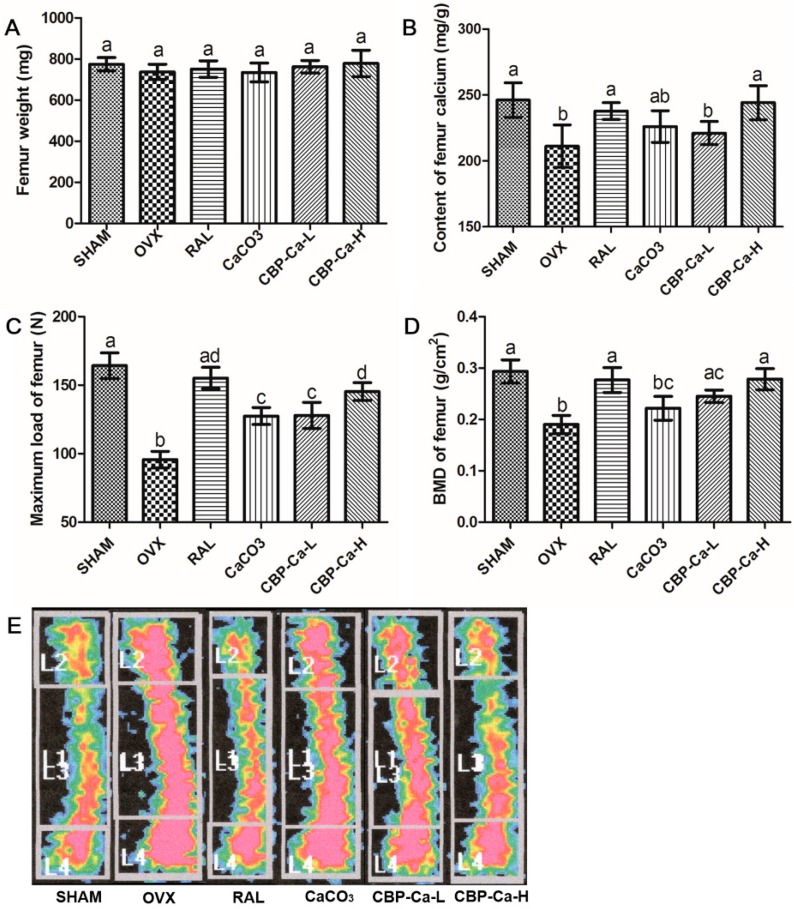
Effects of CBP-Ca on bone properties (*n* = 7/group). The rats were treated for 12 weeks, then their femurs and tibias were dissected for measurements of the weight of the right femurs (**A**), the calcium content of the right femurs (**B**), the bone biomechanical properties of the left femurs (**C**), the BMD of the right tibias (**D**), and the bone mineralization degree of the right tibias (**E**). Different lowercase letters denote statistically significant differences (*p* < 0.05).

Bone parameters including weight, calcium content, maximum load, BMD, and bone mineralization degree were investigated. As shown in Figure 6A, there was no significant difference in femur weight between each group. Figure 6B revealed that the femur calcium content of the OVX group was significantly (*p* < 0.01) lower than that of the SHAM group. Whereas, the femur calcium content was partly recovered by CaCO_3_ (200 mg Ca^2+^/kg/body weight) and low dose CBP-Ca (100 mg Ca^2+^/kg/body weight), and fully recovered by raloxifene and high dose CBP-Ca (200 mg Ca^2+^/kg/body weight).

Ovariectomy strongly induces postmenopausal osteoporosis and bone loss, then leads to poor bone mechanical properties [38]. Mechanical tests indicated that the femur maximum load of the OVX group was significantly (*p* < 0.01) reduced to 58.23% when compared with the SHAM group (Figure 6C). However, it recovered to 94.48%, 77.59%, 77.87%, and 88.44% in RAL, CaCO_3_, CBP-Ca-L, and CBP-Ca-H groups, respectively. This suggested that CBP-Ca could effectively strengthen the mechanical properties of OVX rats [39,40].

BMD was widely recognized as the most preferred standard for the judgment of bone loss and osteoporosis [38,39]. The tibia BMD of OVX group was dramatically decreased to 64.78% compared with the normal rats in the SHAM group (*p* < 0.01) (Figure 6D). However, the BMD of the RAL group, CBP-Ca-L group, and CBP-Ca-H group significantly recovered to 94.34%, 83.53%, and 94.89%, respectively while there was no significant difference between the OVX and CaCO_3_ group. A similar trend was observed in the bone mineralization degree results (see Figure 6E). These suggested that CBP-Ca is superior to CaCO_3_ in preventing osteoporosis. The results indicated that CBP-Ca could improve the bone calcium and BMD, strengthen bone mechanical properties, and thus ameliorate the OVX induced osteoporosis.

### 3.7. CBP-Ca Reduces the Bone Turnover Rate of OVX Rats

Bone turnover (including bone formation and resorption phase) were regulated by the bone remodeling process, and any disturbance in this process will lead to osteopathic disease [24,41]. The postmenopausal osteoporosis was recognized as high bone turnover rate, which was characterized by the increased bone turnover markers [5,26].

**Figure 7:**
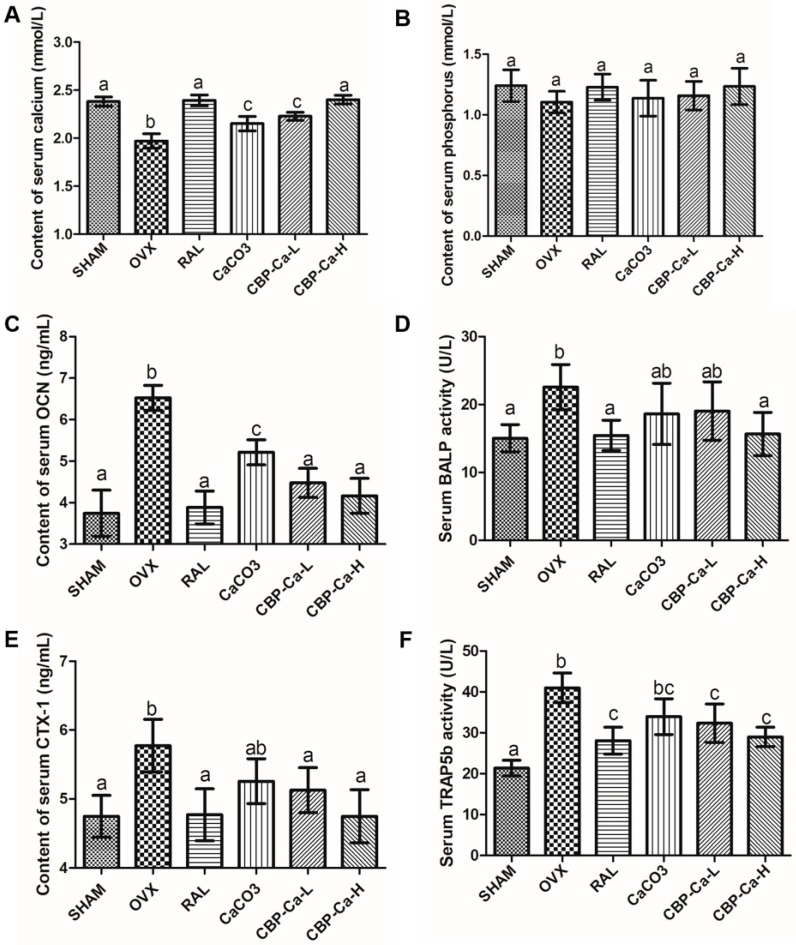
Effects of CBP-Ca on bone turnover markers of OVX rats (*n* = 7/group). All rats were treated for 12 weeks, and the blood was collected. Then the serum was separated for measurements of the content of serum calcium (**A**) and phosphorus (**B**), osteocalcin (OCN) (**C**), the serum bone alkaline phosphatase (BALP) activity (**D**), the content of C-telopeptide of type I collagen (CTX-1) (**E**), and the tartrate-resistant acid phosphatase isoform 5b (TRAP5b) activity (**F**). Different lowercase letters denote statistically significant differences (*p* < 0.05).

Serum Ca, P, OCN, CTX-1, BALP, and TRAP5b are the most important bone turnover markers used in the evaluation of bone formation and resorption [3]. Changes in these serum biochemical markers are shown in Figure 7A–F. Results recorded an insignificant difference in the serum P content between each group (Figure 7B). Whereas, there was a significant decrease in the content of serum Ca, and significant increases in serum OCN, BALP, CTX-1, and TRAP5b of the OVX group when comparing with the SHAM group. These changes were ameliorated by the treatment of RAL, CBP-Ca or CaCO_3_, and the most amelioration was obtained in the RAL and CBP-Ca-H group.

The serum calcium content of the OVX group decreased 17.28%, compared with the SHAM group (Figure 7A). However, the administration of CBP-Ca (200 mg Ca^2+^/kg/body weight) markedly increased the serum Ca to 100.80%. This might result from the outstanding calcium bioavailability of CBP-Ca, according to the calcium absorption results given in Figure 3 and Table 2. A similar result was reported by Alkhamees and co-workers [37].

Serum OCN and BALP, specifically secreted by osteoblasts, are widely considered as important bone formation markers [5]. As seen in Figure 7C,D, compared with the SHAM group, serum OCN and BALP were significantly increased to 168.38% and 150.19% in the OVX group, respectively. The increased levels of OCN and BALP were also found in men with insufficient calcium intake [42] as well as the patients with calcium malabsorption [43]. However, the administration of low dose CBP-Ca significantly (*p* < 0.01) reduced the levels of OCN (31.60% reduction) and BALP (15.62% reduction), in comparison with the OVX group. Treatments with raloxifene, CaCO_3_, and high dose CBP-Ca show similar trends. Wang et al., reported that the Antarctic krill derived peptides could decrease the levels of bone formation markers in OVX rats [26]. Also, Sato et al. suggested that the milk intake could reduce bone formation markers [42]. They are in accordance with the results in the present study.

The serum CTX-1 content and TRAP5b activity were investigated to provide the information of bone resorption degree (see Figure 7E,F). TRAP5b is the enzyme produced by osteoclasts during bone resorption phase [3,24]. As seen in Figure 7F, the serum TRAP5b activity was significantly increased (*p* < 0.01) to 191.93% in the OVX group compared with the normal SHAM group, indicating an OVX-induced increase of osteoclasts activity and excessive bone resorption [24,42]. The serum TRAP5b activity was markedly reduced (*p* < 0.01) by the administration of CBP-Ca, while the CaCO_3_ treated group showed no significant change (*p* > 0.05) compared to the OVX group. A similar phenomenon was observed in CTX-1, the breakdown product of bone type I collagen during bone resorption, which is well recognized as the biochemical marker of osteoclast activity. Similar results were reported by previous studies [24,42]. Results indicated that the bone turnover rate of OVX rats was remarkably (*p* < 0.01) reduced by the treatment of CBP-Ca.

Previous study reported that, in order to maintain the calcium homeostasis, the bone turnover process could be accelerated by a decrease of serum calcium content [35,41] and also sufficient calcium intestinal absorption is critical for reduction of bone turnover rate and alleviation of osteoporosis [2]. Our results indicated that CBP-Ca could significantly increase the intestinal absorption, bioavailability of calcium, and serum calcium, then decrease bone turnover rate, and thus ameliorate osteoporosis.

## 4. Conclusions

In this study, calcium binding peptides (CBP) were prepared from Pacific cod bone. Structural analyses suggested that the –COOH and –NH_3_ groups in CBP could bind with calcium ions and form CBP-Ca. The in situ SPIP experiment indicated that the CBP-Ca could significantly enhance the intestinal absorption and permeability of calcium. Furthermore, in vivo tests in the OVX rat model revealed that CBP-Ca could efficiently improve calcium bioavailability, bone mass, bone mineral density, bone strength, and bone mineralization degree. The mechanism underlying the anti-osteoporosis activity of CBP-Ca might relate to the reduction of bone turnover rate (supported by the decrease of bone turnover markers), resulting from the increase of calcium intestinal absorption and bioavailability. Results indicated that the calcium binding peptides from Pacific cod bone could be employed as functional supplementation in preventing OVX-induced osteoporosis.

## Figures and Tables

**Table 1:** Amino acid composition of calcium binding peptides (CBP) (residues/1000 total amino acid residues).

Amino Acid	CBP	Amino Acid	CBP
Aspartic acid (Asp)	84	Leucine (Leu)	11
Threonine (Thr)	22	Tyrosine (Tyr)	3
Serine (Ser)	59	Phenylalanine (Phe)	10
Glutamic acid (Glu)	97	Lysine (Lys)	14
Glycine (Gly)	322	Histamine (His)	29
Alanine (Ala)	86	Arginine (Arg)	46
Cysteine (Cys)	15	Proline (Pro)	106
Valine (Val)	10	Hydroxyproline (Hyp)	72
Methionine (Met)	2	Imino acid (Pro + Hyp)	178
Isoleucine (Ile)	12	Total	1000

**Table 2:** Effect of CBP-Ca on calcium bioavailability in ovariectomized (OVX) rats.

	Ca Intake (mg/day)	Urinary Ca (mg/day)	Fecal Ca (mg/day)	Ca Apparent Absorption (mg/day)	Ca Apparent Absorption Rate (%)	Ca Retention (mg/day)	Ca Retention Rate (%)
SHAM	57.74 ± 4.34 ^a^	1.48 ± 0.28 ^ac^	19.57 ± 2.90 ^a^	38.17 ± 5.37 ^ac^	65.90 ± 5.97 ^a^	36.69 ± 5.37 ^a^	63.33 ± 6.15 ^a^
OVX	58.95 ± 4.15 ^a^	1.43 ± 0.28 ^ac^	34.63 ± 3.07 ^b^	24.32 ± 4.84 ^b^	41.02 ± 6.41 ^b^	22.96 ± 4.76 ^b^	38.72 ± 6.41 ^b^
RAL	58.18 ± 3.58 ^a^	1.26 ± 0.21 ^ac^	19.43 ± 2.08 ^a^	38.75 ± 2.53 ^a^	66.64 ± 2.56 ^a^	37.49 ± 2.58 ^a^	64.46 ± 2.64 ^a^
CaCO_3_	57.14 ± 3.80 ^a^	1.53 ± 0.09 ^a^	29.13 ± 3.48 ^b^	28.01 ± 5.16 ^bc^	48.79 ± 7.30 ^b^	26.48 ± 5.13 ^b^	46.11 ± 7.32 ^b^
CBP-Ca-L	29.53 ± 2.11 ^b^	0.62 ± 0.10 ^b^	6.39 ± 2.10 ^c^	23.13 ± 2.25 ^b^	78.44 ± 6.64 ^c^	22.51 ± 2.26 ^b^	76.34 ± 6.68 ^c^
CBP-Ca-H	61.70 ± 3.99 ^a^	1.10 ± 0.16 ^c^	18.16 ± 3.34 ^a^	43.54 ± 5.60 ^a^	70.41 ± 5.65 ^c^	42.44 ± 5.65 ^a^	68.62 ± 5.77 ^ac^

All values are presented as mean ± SD (*n* = 7/group). Different lowercases letters in the same column denote significant differences (*p* < 0.05).
